# Environmental Exposures and Asthma Development: Autophagy, Mitophagy, and Cellular Senescence

**DOI:** 10.3389/fimmu.2019.02787

**Published:** 2019-11-29

**Authors:** Karan Sachdeva, Danh C. Do, Yan Zhang, Xinyue Hu, Jingsi Chen, Peisong Gao

**Affiliations:** ^1^Johns Hopkins Asthma & Allergy Center, Johns Hopkins University School of Medicine, Baltimore, MD, United States; ^2^Department of Respiratory Medicine, Xiangya Hospital, Central South University, Changsha, China; ^3^Department of Dermatology, Children's Hospital, Chongqing Medical University, Chongqing, China

**Keywords:** oxidative stress, autophagy, mitophagy, senescence, asthma

## Abstract

Environmental pollutants and allergens induce oxidative stress and mitochondrial dysfunction, leading to key features of allergic asthma. Dysregulations in autophagy, mitophagy, and cellular senescence have been associated with environmental pollutant and allergen-induced oxidative stress, mitochondrial dysfunction, secretion of multiple inflammatory proteins, and subsequently development of asthma. Particularly, particulate matter 2.5 (PM_2.5_) has been reported to induce autophagy in the bronchial epithelial cells through activation of AMP-activated protein kinase (AMPK), drive mitophagy through activating PTEN-induced kinase 1(PINK1)/Parkin pathway, and induce cell cycle arrest and senescence. Intriguingly, allergens, including *ovalbumin* (OVA), *Alternaria alternata*, and *cockroach allergen*, have also been shown to induce autophagy through activation of different signaling pathways. Additionally, mitochondrial dysfunction can induce cell senescence due to excessive ROS production, which affects airway diseases. Although autophagy and senescence share similar properties, recent studies suggest that autophagy can either accelerate the development of senescence or prevent senescence. Thus, in this review, we evaluated the literature regarding the basic cellular processes, including autophagy, mitophagy, and cellular senescence, explored their molecular mechanisms in the regulation of the initiation and downstream signaling. Especially, we highlighted their involvement in environmental pollutant/allergen-induced major phenotypic changes of asthma such as airway inflammation and remodeling and reviewed novel and critical research areas for future studies. Ultimately, understanding the regulatory mechanisms of autophagy, mitophagy, and cellular senescence may allow for the development of new therapeutic targets for asthma.

## Introduction

Asthma is a leading serious chronic illness of children and adults worldwide, and its prevalence has been increasing over the past few decades (1, 2). Million people worldwide are affected, including 24 million in the United States (3). Asthma is chronic airway inflammation characterized by airway hyper-responsiveness, wheezing, cough, and dyspnea, and has become a major contributing factor to missed time from school and work, and is also a major cause of hospitalization and emergency department visits. It is well-recognized that the increase in asthma prevalence may be mainly attributed to industrialization- and urbanization-generated environmental pollutants (4–10). In China, a study of over 30,000 adults showed that the prevalence of persistent cough, sputum production, and wheezing was associated with major traffic roads, factories, and large smokestacks (11). This was supported by another cross-sectional study of 23,326 Chinese children, which showed that the prevalence of asthma was higher for those residing near areas with serious air pollution (12). Diesel exhaust particles (DEPs) are of particular concern and contributed to more than 90% of the particulate matters (PMs) derived from traffic sources in European and American cities (13). Particulate matter 2.5 (PM_2.5_), one of the major pollutants in urban areas, accounts for a large proportion of the atmospheric particulate matter and increased prevalence and symptom severity in children and adult patients with asthma (14–16) and other respiratory diseases (17, 18). PM_2.5_ as a mixture of various chemical constituents has been shown to promote oxidative stress and inflammation (19). Furthermore, concentrated transition metals in the environment have been shown to stimulate the production of reactive oxygen species (ROS) 19, leading to airway injury and inflammation (20).

In addition to environmental pollutants, it is well-known that environmental allergens are also major players in the development of allergic sensitization and asthma. Importantly, recent studies made novel findings that environmental pollutants co-exposure with allergens can lead to increased allergic sensitization and severe asthma (21–23). Particularly, prenatal exposure to DEPs is associated with an increased risk of allergic sensitization, early childhood wheeze, and asthma (24, 25). Of interest, co-exposure to DEP and house dust mite (HDM) can promote allergic sensitization and induce major features of a more severe asthma (9, 26–29). Furthermore, we have recently shown that benzo(a)pyrene (BaP) co-exposure with dermatophagoides group 1 (Der f 1) can activate aryl hydrocarbon receptor (AhR) signaling, which regulates ROS generation and TSLP and IL-33 expression (30). Similarly, a very recent study demonstrated that PM2.5 disturbs the balance of Th17/Treg cells by impairing differentiation of T_reg_ cells and promoting differentiation of Th17 cells through the molecular pathways AhR–HIF-1α (hypoxia-inducible factor-1alpha) and AhR–Got1 (glutamate oxaloacetate transaminase 1) in a cockroach allergen-induced mouse model of asthma (31). Warren et al. reported that acute inhalant exposure to an agriculture acquired organic dust extract (ODE) impacts lung inflammatory responses in a murine model of experimental allergic asthma, suggesting that allergic asthma may prime the lung microenvironment response toward an exaggerated response following exposure to a dusty farm environment (32). Thus, future studies are warranted to identify the underlying mechanisms regarding the co-exposure-induced exacerbation of allergic asthma. In this review, we evaluated the literature regarding the basic cellular processes, including autophagy, mitophagy, and cellular senescence, and discussed their involvement in environmental pollutant/allergen-induced major features of asthma and biological regulation. Additionally, we identified areas of unmet research needed and their potentials as novel therapeutic avenues for the treatment of asthma and allergic diseases.

## Autophagy

It has been postulated that dysregulation of basic cellular processes which maintain homeostasis and physiological balance may lead to the key clinical features of asthma. Autophagy, a homeostatic process with multiple effects on immunity, has been shown to play important roles in causing downstream changes initiated by environmental pollutants, allergens, and respiratory tract infections (33–40). Autophagy is a mechanism in which the eukaryotic cell encapsulates damaged proteins or organelles for lysosomal degradation and recycling (41). The autophagic pathway has recently been suggested to be involved in the several key features of asthma pathogenesis, including eosinophilic airway inflammation (42), airway hyper-responsiveness (36), and airway remodeling (43). It has been shown that PM_2.5_ exposure can induce cell autophagy and airway inflammation through different immunological and molecular mechanisms (44–46). Furthermore, exposure to allergens has also been shown to activate autophagy, as demonstrated in studies with cockroach allergen (47), *Alternaria* extract (48), and caffeine (49).

Autophagy is a process that has been maintained over ages of evolution, and by which damaged and misfolded proteins along with aged or damaged organelles are transported to lysosomes for elimination and digestion (50). Currently, three major types of autophagy are recognized: macroautophagy, microautophagy, and chaperone-mediated autophagy (51). Of these, macroautophagy is the most extensively studied, which uses autophagosomes, double-membraned vesicles, to engulf cytoplasmic proteins and organelles for delivery to the lysosome for degradation. Autophagosomes fusing with lysosomes are termed autophagolysosomes (52). After fusion with lysosomes, the cargo delivered is degraded by lysosomal enzymes and then transported to the cytoplasm (53–55). The byproducts of lysosomal degradation (e.g., amino acids) are recycled and then used for protein synthesis that enables salvage of energy normally used in *de novo* synthesis. Microautophagy as a second type of autophagy does not require autophagosomes but involves the direct engulfment of the cargo that may include proteins and lipids by the invagination of the lysosomal membrane (56). Chaperone-mediated autophagy (CMA) as a third type of autophagy is unique to mammalian cells (57). CMA is a highly regulated cellular process that involves the degradation of a selective subset of cytosolic proteins in lysosomes. In contrast to macroautophagy that engulfs and delivers predominantly larger structures for bulk degradation of cargo, CMA delivers individual proteins for lysosomal degradation. CMA involves a co-chaperone complex led by heat shock cognate 70 (HSC70) that recognizes target proteins that have a KFERQ-like pentapeptide sequence (52). Chaperone-bound proteins are transported to lysosomes, in which they are recognized by the lysosome-associated membrane protein type 2a (LAMP2a) receptor, a major regulator of CMA. LAMP2a is a transmembrane protein component that oligomerizes and forms a translocon complex for internalization and degradation of chaperone-delivered cargo in the lysosome (58). In this review, we mainly focused on macroautophagy, the form of autophagy dealing with the destruction and recycling of damaged macromolecules and organelle structures, and highlighted the significance of macroautophagy in the maintenance of cellular energetic balance and homeostasis.

## Regulation of Autophagy

**Figure 1:**
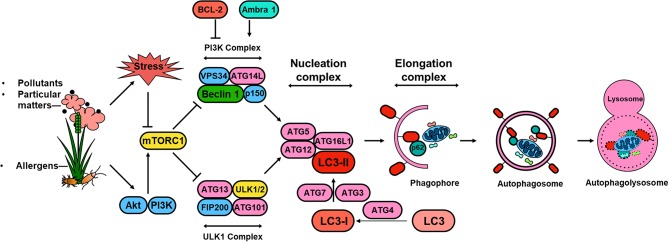
Schematic overview of autophagy regulation. Environmental signals, such as environmental pollutants and allergens, induce cellular stress leading to the activation of the mTOR signaling complex 1 (mTORC1). Induction of autophagy begins with the formation of the phagophore, which is initiated by the ULK complex, consisting of ULK1 (or ULK2), autophagy-related protein 13 (ATG13), FAK family kinase interacting protein of 200 kDa (FIP200) and ATG101. PI3K complex, consisting of the vacuolar protein sorting 34 (VPS34) and the regulator subunits ATG14L, p150 and beclin 1, provides further nucleation signal. Autophagosome formation requires phagophore membrane elongation by a complex composed of ATG5, ATG12, ATG16L, and LC3-II, which are derived from the microtubule-associated protein 1 light chain 3 (LC3) by the activity of ATG4 generating LC3-I and the conjugation C-terminal glycine of LC3-I to phosphatidylethanolamine by ATG7, and ATG3. The formation of the autophagolysosome is a result of the fusion between the autophagosome and lysosomal compartments. Lysosomal hydrolyases degrade the autophagy cargo in all three processes.

Significant progress has been made in understanding the molecular mechanisms of autophagy and the regulation of autophagy in the past 10 years (59). These studies, together with discoveries of the autophagy-related (ATG) genes and their associations with specific diseases (60, 61), provide a multidimensional perspective of mechanisms by which ATG gene-dependent autophagy pathways are critical in the pathogenesis of human diseases. The autophagy pathway is usually described as involving a set of 16–20 core conserved ATG genes. These core proteins are involved in regulating initiation of autophagy by the UNC51-like kinase (ULK) complex (e.g., ULK1, FIP200, ATG13), autophagosome nucleation (Beclin 1, VPS34, VPS15, and ATG14), autophagosome elongation and maturation (e.g., ATG5, ATG12, ATL16L1, ATG8/microtubule-associated protein 1 light chain 3 [LC3]), and induction of autophagosomes and fusion of autophagosomes with lysosomes (i.e., ATG9/mammalian Atg9 and vacuole membrane protein 1) (59, 62). Amongst these ATG proteins, LC3 is a well-defined protein, which is cleaved from a pro-form by Atg4 and then conjugated with phosphatidyl-ethanolamine by the sequential action of Atg7 and Atg3 (63) to form LC3-II (Figure 1). The conversion of LC3-I (unconjugated cytosolic form) to LC3-II (autophagosomal membrane-associated phosphatidylethanolamine-conjugated form) has been considered as a major feature of autophagosome formation. Additionally, SQSTM1/p62 has an ubiquitin binding domain and an LC3 interaction domain and thus can bring ubiquitinated cargos to the autophagosomes for autophagy.

Significant numbers of signaling molecules particularly cytokine have been shown to regulate autophagy (52, 64). For example, IL-10 and IL-10 receptor signaling inhibits the starvation induced autophagy of murine macrophages via class I phosphatidylinositol 3-kinase (PI3K) pathway (64), suggesting that IL-10 plays a critical role in the autophagic process of macrophages. Distinct classes of PI3K have previously been shown to be involved in signaling pathways that control macro-autophagy in human colon cancer HT-29 cells (65, 66). Moreover, the Th1 cytokine IFN-gamma induces autophagy in macrophages (67). In contrast, Th2 cytokines, IL-4 and IL-13, inhibit autophagy in macrophages under starvation or IFN-gamma stimulation, and inhibit autophagy-mediated killing of intracellular mycobacteria in murine and human macrophages (68). Intriguingly, recent studies suggest that IL4 can induce autophagy in activated CD4^+^ Th2 cells (68), primary dendritic cells (DCs) (69), and primary B cells that exacerbates experimental asthma through different mechanisms (70). Similarly, IL-13 alone can activate autophagy in airway epithelial cells and drive the secretion of excess mucus (71). These findings suggest that Th2 cytokines may play a dual role in autophagy induction depending on different cell types. However, further studies are essential to investigate how differential modulation of autophagy by Th1 and Th2 cytokines in different cell types, which may represent a key feature of the host response to environmental stresses. Furthermore, neutralization of the receptors VEGFR, β-integrin or CXCR4, or IL-10 can also regulate autophagy by restoring autophagy in macrophage/monocytic cells exposed to HIV-1-infected cells (72). In contrast, autophagy can also regulate cytokine production (73). For example, Atg16L1 is an essential component of the autophagic machinery responsible for control of the endotoxin-induced IL-1β production (74). It has also been shown that autophagy influences IL-1β secretion by either targeting pro-IL-1β for lysomal degradation or regulating activation of the NLRP3 inflammasome (73). Similarly, autophagy plays a pivotal role in the induction and regulation of IL-23 secretion and innate immune responses through effects on IL-1 secretion (75). Furthermore, autophagy regulates inflammatory cytokine secretion [e.g., macrophage migration inhibitory factor (MIF)] by macrophages through controlling mitochondrial ROS (76). ROS can activate STAT3 transcriptional factor, leading to the secretion of IL-6 in starvation-induced autophagy of cancer cells (77). Interestingly, *Alternaria* extract as a major outdoor allergen can activate autophagy that subsequently induces IL-18 release from airway epithelial cells (48).

In addition to cytokines, several significant molecules have also been identified to regulate autophagy (52). Of these, mTOR (mammalian target of rapamycin) has been shown to regulate cell-signaling pathways after exposure to several major factors including amino acids, oxidative stress, energy levels, and growth factors (78, 79). Particularly, mTORC1 (one of the functional forms of mTOR) regulates autophagy by directly interacting with the ULK complex ULK1-ATG13-FIP200 (80). mTORC1 can suppress autophagy by inhibiting ATG1/ULK complexes under normal physiological conditions (51). In addition, AMP-activated protein kinase (AMPK)/ULK1 pathway mediates autophagy by transmitting stress signals for autophagosome formation, independent of mTOR signaling (80, 81). AMPK is capable of inhibiting non-autophagy VPS34 complexes but activating the proautophagy VPS34 by the phosphorylation of Beclin 1 (Beclin1/VPS34) to initiate phagophore formation (82). In addition to AMPK and mTORC1, calmodulin-dependent protein kinase II (CaMKII) also plays a role in engaging autophagy regulation (83). CaMKII, a serine/threonine-specific protein kinase regulated by the Ca^2+^/calmodulin complex, can directly phosphorylate Beclin 1 at Ser90 that enhances K63-linked ubiquitination of Beclin 1 and activation of autophagy (84). CaMKII can also stimulate K63-linked ubiquitination of inhibitor of differentiation 1/2 (Id-1/2). Of interest, the increased ubiquitinated Id-1/Id-2 can bind p62 and then be transported to autolysosomes for degradation, which can subsequently promote the differentiation of neuroblastoma cells and suppress the proportion of stem-like cells (84).

Recently, transcriptional regulation of autophagy genes has drawn a lot of attention in autophagic responses to specific stimuli (85). Several transcription factors and histone modifications have been identified to regulate autophagy gene expression. In addition to the well-known two transcription factors, p53 and Forkhead box O3 (FOXO3) (86), Transcription Factor EB (TFEB) is one of the most recently identified transcriptional regulators of autophagy (87). TFEB is highly phosphorylated by various kinases such as AKT, Extracellular Signal-Regulated Kinase 2 (ERK2), and mTORC1, and sequestered in the cytoplasm under nutrient rich conditions. In contrast, TFEB is dephosphorylated by calcineurin (CaN) and translocates to the nucleus where it activates autophagy and lysosome gene transcription upon nutrient deprivation (88). Forkhead box K (FOXK) engages in the transcriptional repression of autophagy gene expression by binding to promoter regions of early-stage autophagy genes (e.g., ULK complex) and recruits the SIN3A-Histone deacetylase (HDAC) repressor complex to these regions under nutrient rich conditions (89). However, most intriguingly, the post-translational modification status on histones is also linked to autophagy gene regulation, including histone H4K16 acetylation, H3K9 dimethylation, and H3K27 trimethylation (90). Of these, H4K16 acetylation suppresses autophagy gene expression through H4K16 acetyltransferase human Males absent On the First (hMOF) degradation and/or Sirtuin1 (SIRT1)-dependent histone deacetylation (91). H3K27 trimethylation catalyzed by Enhancer of Zeste Homolog 2 (EZH2) suppresses the expression of negative regulators of the mTORC1 signaling components and leads to mTORC1 activation and autophagy inhibition (92). Interestingly, many of the transcriptional factors that modulate expression of autophagy genes are regulated by common upstream kinases such as mTORC1 and AMPK. Furthermore, histone modification status is also a significant determinant of transcriptional regulators to autophagic stimuli.

## Autophagy and Key Features of Asthma

**Figure 2:**
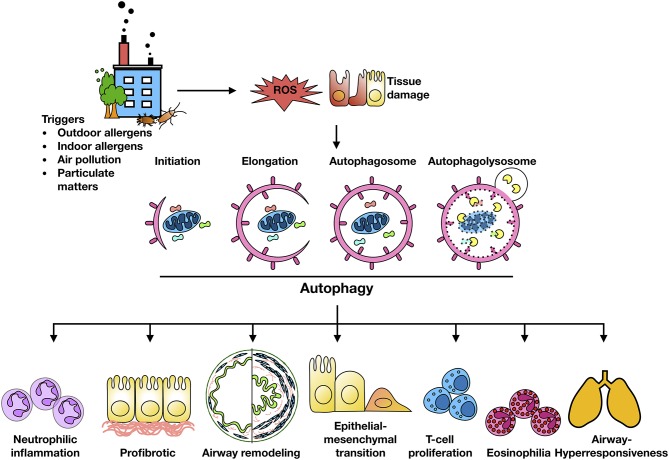
Autophagy and major features of asthma. Environment/allergen exposure initiates ROS generation in airway epithelial cells, which serve as “signaling molecules” modulating the process of autophagic cycle through activating signaling molecules and autophagy pathways, thereby leading to the major phenotypic changes of asthma, including airway inflammation, airway remodeling, and airway hyper-responsiveness.

Exposure to traffic and industrial pollution particulate matters, predominantly DEPs, have been shown to increase the risk of asthma (15, 26). Environmental pollutants (e.g., PM_2.5_) can induce ROS generation and impair lung function in asthmatic patients (93–97). It was well-documented that ROS are key mediators that contribute to oxidative damage and chronic airway inflammation in allergy and asthma (98–101). However, the underlying mechanisms still remain unclear. Recent studies have suggested that autophagy may be a new frontier in human asthma (50) and may play a crucial role in chronic airway inflammation (42). Indeed, higher autophagy levels have been shown in sputum granulocytes, peripheral blood cells and peripheral eosinophils of patients with severe asthma (102). The increased autophagy has been associated with important immune mechanisms and extracellular matrix deposition and fibrosis in airway remodeling in asthma (43). Furthermore, genetic mutations in autophagy genes have been associated with asthma. For example, single nucleotide polymorphisms in *Atg5* are correlated with reduced lung function (103). Thus, these cumulative findings raise the possibility that environment/allergen exposure initiates the production of ROS in airway epithelial cells, which serve as “signaling molecules” modulating the process of autophagic cycle through activating signaling molecules and autophagy pathways, thereby leading to the major phenotypic changes of asthma as summarized in Figure 2, including airway inflammation, airway remodeling, and airway hyper-responsiveness.

## Autophagy and Airway Inflammation

Autophagy plays important roles in airway inflammation (36, 42). It has been suggested that autophagy plays a critical role in PM-induced inflammation in airway epithelium through the activation of NF-kB1 and activator protein-1 (AP-1) (104). Furthermore, PM_2.5_ can induce inflammatory cytokine release (e.g., IL-6, IL-8, IL-1β1, and TNFα) and oxidative injury of lung cells (105). Additionally, Long et al. found that PM_2.5_ can induce cell arrest in the G0/G1 phase and increase mitochondrial membrane potential, ROS generation, and airway epithelial cell apoptosis (106). PM_2.5_ not only induced the production of pro-inflammatory cytokine IL-6, TNFα, and activation of AMPK, but also promoted the expression of ATG5, Beclin-1 and LC3II in the airway epithelial cells (107). Interestingly, knockdown of ATG5 limited PM_2.5_ -induced autophagy, ROS generation, cell apoptosis, and production of IL-6 and TNFα. Mechanistically, this study suggests that the activation of AMPK may be critical in autophagy-mediated PM_2.5_-induced airway inflammation. In addition, allergens have also been shown to induce autophagy. OVA (ovalbumin) used in a murine asthma model can induce autophagy in airway tissues (36). *Alternaria alternata* as one of the major outdoor allergens that cause allergic airway diseases (108) has been shown to induce IL-18 secretion from airway epithelial cells, and thereby initiate Th2-type responses (109). IL-18 is a pro-inflammatory cytokine that belongs to the IL-1 family (110). Importantly, *Alternaria* extract stimulation can activate an autophagy-based unconventional secretion pathway and induce airway epithelial cells to release IL-18 via an autophagy dependent, but caspase 1 and 8 independent pathway (48). Studies from our research group showed that cockroach extract can induce autophagy in airway epithelial cells *in vitro* and in a mouse model of asthma (47). Further studies on the underlying mechanisms demonstrated that ROS and oxidized CaMKII (ox-CaMKII) in airway epithelial cells are critical in regulating cockroach allergen-induced autophagy (111).

Although environmental pollutants/allergens can induce autophagy, its role in airway inflammation remains unclear. It has been suggested that, at baseline, autophagy is critical for inhibiting spontaneous lung inflammation and is fundamental for airway mucus secretion by airway goblet cells. Autophagy deficient mice (Atg5^−/−^ and Atg7^−/−^) develop spontaneous sterile lung inflammation (110). Similarly, deficiency of CD11c-specific autophagy results in severe IL-17A-mediated neutrophilic lung inflammation and unprovoked spontaneous airway hyperactivity (112). Furthermore, deficiency of ATG5 in airway epithelial cells results in an increased airway inflammation (113), and disruption or deletion of autophagy in airway epithelial cells resulted in airway hyperreactivity (114). Autophagy deficiency (ER-Cre: *Atg7*^*fl*/*fl*^) in mice after exposure to *P. aeruginosa* impairs pathogen clearance, increases neutrophilic inflammation, and the production of IL-1β (115). Although autophagy appears to be a protective mechanism, autophagy may also exacerbate airway inflammation. For example, inhibition of autophagy by 3-MA and intranasal knockdown of Atg5 led to marked improvement in AHR, eosinophilia, IL-5 levels in bronchoalveolar lavage fluid, and histological inflammatory features (36). Similarly, autophagy deficiency in macrophages (siRNA targeting PIK3C3) during LPS-induced lung inflammation attenuates lung and bronchoalveolar immune cell infiltration and air space cytokine levels (116). Additionally, IL-4-induced autophagy in B cells exacerbated asthma through an mTOR-independent, PtdIns3K-dependent pathway (70). Thus, autophagy may play diverse roles, either protective or detrimental, in asthma. Although the reason is unknown, it has been suggested that autophagy may represent a protective role in maintaining homeostasis at baseline or during acute infection, but play a detrimental role due to impaired autophagy or a persistent autophagy responses leading to an accumulation of excessive autophagosome in a prolonged exposure of environmental pollutants/allergens or inflammation. Furthermore, autophagy involvement in different cell types may result in different characteristic phenotypic changes. For example, deletion of ATG5 and ATG14 or pharmacological inhibition (e.g., 3-MA, Baf-A1) in cultured airway epithelial cells treated with IL-13 results in less mucus secretion and less CCL26 secretion. In contrast, autophagy deficiency in macrophages (117) or DCs (112) results in the exacerbation of inflammation. Thus, the investigation of the real impact of autophagy, protective or detrimental, is extremely challenging.

## Autophagy and Airway Remodeling

Recent studies have linked autophagy to the major features of airway remodeling in asthma, including airway smooth muscle (ASM) 44 (118–120), extracellular matrix (ECM) (121, 122), fibrosis (117), and epithelial-mesenchymal transition (EMT) (123). Particularly, it has been suggested that TGFβ1 induced autophagy is essential for collagen and fibronectin production in human airway smooth muscle cells, and deletion of Atg5 and Atg7 leads to reduction in pro-fibrotic signaling and ECM protein release (50, 124). In turn, autophagy has also been shown to participate in profibrotic changes induced by TGFβ1 (125). Furthermore, McAlinden et al. provided evidence of increased activation of the autophagy pathway in the airways of patients with asthma (43). Especially, they showed an association for TGFβ1 and accumulation of collagen and increased profibrotic signaling in an autophagy-dependent manner in ASM cells (43). Furthermore, inhibition of autophagy in murine model has been shown to attenuate airway inflammation and reduce the concentration of TGFβ1, and subsequently lead to a reduced airway remodeling. However, the critical mechanistic evidence is limited.

## Mitophagy

**Figure 3:**
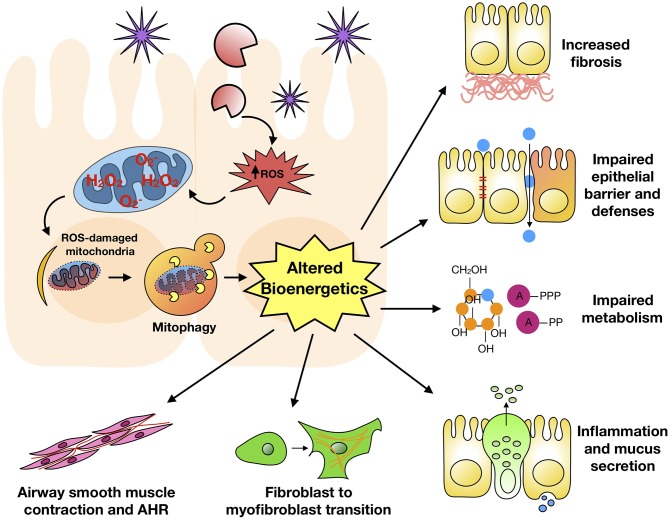
Mitophagy and major features of asthma. Environmental pollutant/allergen induces excessive ROS generation, mitochondrial dysfunction, and mitophagy, subsequently leading to dysregulation of mitochondrial bioenergetics. These may ultimately result in significant phenotypic changes observed in asthma.

Mitophagy is the selective degradation of mitochondria by autophagy. It often happens to damaged mitochondria following the exposure to environmental pollutants/allergens or stress and plays a critical role in promoting turnover of mitochondria and preventing accumulation of dysfunctional mitochondria (38). Mitochondrial dysfunction and elevated ROS production have been associated with allergic diseases, including atopy, atopic dermatitis, and asthma (126–129). Of interest, a disturbance in the homeostasis of mitochondria leads to ROS generation, which cause weakened barriers and subsequently airway inflammation, epithelial fragility, and impaired secretion capacity (130). Furthermore, PM_2.5_-exposed rat lung injury is associated with mitochondrial fusion-fission dysfunction, mitochondrial lipid peroxidation and cellular homeostasis imbalance, and ROS generation, leading to the disruption of mitochondrial dynamics (131). PM_2.5_ can regulate the dynamics of mitochondria via facilitating mitochondrial fission, and the excess ROS induced by PM_2.5_ can trigger mitophagy by activating PINK1/Parkin pathway (132). Acrolein, an ubiquitous environmental pollutant that is abundant in tobacco smoke, cooking fumes, and automobile fumes (133), has also been reported to induce mitochondrial DNA (mtDNA) damages, mitochondrial fission and mitophagy in human lung cells (134). Of interest, mitophagy was found to prevent mitochondria-induced inflammation (mito-inflammation) (135). Thus, mitophagy may be critical in environmental pollutant/allergen-induced mitochondrial dysfunction and dysregulation of mitochondrial bioenergetics. These may ultimately result in a dysregulated mitophagic cycle and significant phenotypic changes observed in asthma (Figure 3).

## Molecular Mechanisms of Mitophagy

Mitophagy is an evolutionarily conserved homeostatic process by which the cells selectively degrade only dysfunctional or damaged mitochondria (136, 137). Mitophagy is a normal physiological process during cell life and functions as surveiling mitochondrial population, eliminating superfluous and/or impaired organelles (137). Defective removal of damaged mitochondria leads to hyper-activation of inflammatory signaling pathways and subsequently to chronic systemic inflammation. However, important questions remain regarding the molecular mechanisms of mitophagy. It has been suggested that mitophagy is regulated by “PTEN-induced kinase 1 (PINK)-Parkin-mediated pathway” and “receptor-mediated pathway” (137). Under physiological conditions, the transport of PINK1 preprotein onto the inner mitochondrial membrane (IMM) is followed by sequential proteolytic cleavage by the mitochondrial processing peptidase and pre-protein-associated rhomboid-like protease (138). Under challenged condition, active PINK1 accumulates on the outer mitochondrial membrane (OMM) through its interaction with the translocations of the outer mitochondrial membrane complex (TOM complex), promoting Parkin recruitment through phosphoralation of both Parkin and ubiquitin (139). In turn, Parkin triggers the polyubiqutination of several OMM proteins, including voltage-dependent-anion-selective channer 1 (VDAC1), mitofusin 1 and 2 (MFN1/2), and mitochondrial import receptor subunit TOM20 homolog (TOMM20) 138. Several adaptor molecules [e.g., p62, optineurin (OPTN), and nuclear domain protein 52 (NDP52)] bindphosphorylated polyubiqutinated proteins and initiate autophagosomal formation through binding with LC3 (140). Recent studies suggest that both NDP5 and OPTN are phosphorylated by the Tank-binding kinase 1 (TBK-1) which enhances their binding affinity to ubiquitin (141). The OPTN-TBK1 complex forms a feed-forward mechanism that speeds up the mitochondrial clearance (142). In contrast, the receptor-mediated mitophagy is dependent on various OMM proteins such as Nip3-like protein X (NIX), BCL2 interacting protein 3 (BNIP3), and Fun14 domain-containing protein 1 (FUNDC 1) (137, 140). These proteins localize to the OMM and interact directly with LC3 to regulate mitochondrial elimination. Cardiolipin and prohibitin 2 (PHB2) are externalized to OMM and interact with LC3 in response to mitochondrial damage to promote the engulfment of defective mitochondria (143, 144).

## Mitophagy and Asthma

Similar to autophagy, accumulating evidence suggests that both enhanced and impaired mitophagy has an important role in the pathogenesis of COPD and lung fibrosis (145–149). However, limited studies were found for asthma. Recent studies have shown that PM_2.5_ can induce increased ROS and mitochondrial damage, which triggers the mitophagy through activating PINK/Parkin pathway (132). In the nucleus, excessive ROS could activate HIF-1 FOXO3, and NRF2, which promote the transcription of BNIP33/NIX, LC3/BNIP3, and p62, thereby facilitate mitophagy (150). Furthermore, the impairment of mitochondrial degradation by mitophagy can lead to the accumulation of fragmented mitochondria and activation of the mitochondrial apoptosis pathway (134). Thus, these studies suggest that environmental pollutants can induce ROS and mitochondrial damage, which triggers mitophagy to maintain stable mitochondrial function in cells by scavenging impaired mitochondria and reducing excessive ROS. Further studies on regulatory mechanisms regarding ROS and mitophagy may provide a new angle on therapies for allergy and asthma.

## Cellular Senescence

Cellular senescence is characterized by irreversible cell cycle arrest and triggered by a number of factors such as aging, DNA damage, oxidative stress, mitochondrial dysfunction (151–153), telomere shortening (154, 155), epigenetic modifications (156), and inflammation (157). Senescence arrest occurs mostly in the G1 phase of the cell cycle, distinguishing it from G0-arrested quiescent cells, and is mediated by cyclin-dependent kinase inhibitors (CDKis) (e.g., p21^CIP1^, p16^INK4a^) and is dependent on the TP53 and pRB tumor suppressor pathways (158). Also, telomeres and nucleoprotein complexes located at the ends of linear chromosomes (159) are critical to the cellular senescence (160). Furthermore, senescent cells accumulated in tissues secrete a large amount of pro-inflammatory mediators termed the senescence associated secretory phenotype (SASP), which drives chronic inflammation, leading to further senescence (157). The composition of the SASP is stimulus-dependent and includes pro- and anti-inflammatory cytokines, chemokines, matrix metalloproteinases, growth factors, and other factors, and has an important role in the immune-mediated clearance of senescent cells and tissue dysfunction (160). Senescent cells have been found at sites of chronic age-related diseases like osteoarthritis (161), atherosclerosis (162–164), and aging lung (153), highlighting the significant role of senescent cells in the pathogenesis of chronic diseases. Senescent cells exhibit increased protein turnover and massive proteotoxic stress due to augmented autophagy and SASP component synthesis (165). Senescence cells also show increased rates of mitochondrial metabolic activity, including the tricarboxylic acid cycle, oxidative phosphorylation, and glycolytic pathways. Senescent cells have increased AMP/ADP:ATP and NAD+/NADH ratios, activating AMPK, which reinforces a TP53-dependent cell-cycle arrest (166, 167). In addition, senescent cells do not proliferate, but are resistant to autophagy and apoptosis, and are thus long living. Importantly, senescent cells can exacerbate mitochondrial dysfunction, inflammation, and other disease-promoting pathways through SASP (153).

Accumulation of senescent cells may slow or stop cell regeneration and tissue maintenance, thus leading to tissue aging (166). Indeed, clearing senescent cells from tissues of mouse models was shown sufficient to delay, prevent, or alleviate multiple age-related disorders (168). Although the underlying mechanisms regarding the elimination of senescent cells are poorly understood, the immune system has been recognized to be critical (169). Different immune cells have been suggested to be involved in the surveillance of senescent cells, including neutrophils, macrophages, natural killer cells, and CD4^+^ T cells (170). These immune cell-derived senescent cells can be immunogenic by expressing stimulatory ligands (e.g., MICA/B) that bind to NKG2D and activating their killing by NK cells (171). Furthermore, senescent cells can recruit immune cells to eliminate senescent cells by secreting cytokine and chemokines (172). Interestingly, recent studies support a balance between activating and inhibitory signals that will determine whether NK and T-cells respond to senescent cells. These studies also suggest a novel mechanism whereby the increased expression of HLA-E on senescent fibroblasts reduced the clearance of senescent cells by NK and CD8^+^ T cells expressing inhibitory receptor NKG2A (157). This represents a novel therapeutic approach to improve the immune clearance of senescent cells by blocking the interaction between HAL-E and NKG2A. In addition, the SASP-related cytokine IL-6 contributes to the increased expression of HAL-E in senescent cells, and that persistent inflammation may result in remaining of senescent cells in tissues, further contributing to the diseases.

## Cellular Senescence and Asthma

Mitochondrial dysfunction has been demonstrated to be able to drive a cell into premature senescence, which affects airway diseases (151, 152). Indeed, the potential role of cell senescence in the pathogenesis of asthma has drawn great attention (162). Studies have implicated that cell senescence in the lung may be an important risk factor for the development of asthma (39, 173). Both COPD and idiopathic pulmonary fibrosis (IPF) are increased in prevalence with age and have been associated with senescence (174, 175). Senescence-related changes are also found in the lungs of adults with asthma, and in the airways of asthmatic children (176). However, mechanistic links between environmental pollutants, allergens, senescence, and pathophysiology of asthma have not been established. Studies have demonstrated that exposure to PM_2.5_ can induce senescence of human dermal fibroblasts (177). Increased exposure to PM_2.5_ is correlated with shortened telomeres in placental tissues and umbilical cord blood (178). Similarly, Bisphenol A (BPA) can induce Th2 inflammatory cascade and trigger DSB-ATM-p53 signaling pathway leading to cell cycle arrest, senescence, autophagy, and stress response in human fetal lung fibroblasts (179). Telomeres are critical to the cellular senescence (160), and telomere shortening is a strong indication of cellular senescence. In a study of 730 mother-baby pairs, increased exposure to PM_2.5_ has been shown to correlate with shortened telomeres in placental tissue and umbilical blood (178). Telomere shortening was also associated with airway hyper-responsiveness and is an inducer of accelerated replicative senescence of bronchial fibroblasts in patients with asthma (154). This was supported by findings in the chronic asthmatic patients who also displayed shorter telomere lengths and suggested that asthma chronicity may be associated with telomere length even at early ages (155). Furthermore, TSLP-induced cellular senescence with elevated p21 and p16 in human epithelial cells was essential for airway remodeling *in vitro* (39). This was further supported by the fact that inhibition of TSLP signaling attenuates epithelial senescence, airway hyper-reactivity, and airway remodeling in an OVA mouse model (39). Plasminogen activator inhibitor (PAI-1), a well-known cell senescence and fibrosis mediator, could activate p53 and mediate bleomycine- and doxorubicin-induced alveolar type II (ATII) cell senescence (180). Further studies suggest that PAI-1 mediates TGF-β1-induced ATII cell senescence, which may contribute to lung fibrogenesis in part by activating alveolar macrophages via secreting pro-fibrotic and pro-inflammatory mediators. Interestingly, this effect is highly dependent on the target cell, because it seems that PAI-1 has opposite effects on fibroblasts and ATII cells in patients with IPF (181). Together, these findings suggest a significant role of senescence in airway fibrosis and remodeling.

Many studies also support the rationale that senescence is associated with a higher pro-inflammatory cytokine profile (182). Of note, higher amounts of IL-6 have been found in patients with asthma and have been shown to trigger or to reinforce premature cellular senescence (183). This IL-6 driven immune-senescence may serve as part of a feed forward loop that drives asthma progression and reduces the efficacy of anti-inflammatory treatments. SASPs released from senescent cells contain inflammatory cytokines that may increase inflammation and impair cellular function in asthma. Furthermore, elevated p21 expression in asthmatic epithelium is not reduced with corticosteroid treatment (40), and in turn, loss of p16^INK4a^ protein results in decreased cell sensitivity to dexamethasone treatment (184), raising the possibility that senescence may play an important role in glucocorticoid resistance in the patients with asthma. Indeed, lymphocyte senescence in COPD has been suggested to be associated with loss of glucocorticoid receptor (GCR) expression by pro-inflammatory/cytotoxic lymphocytes (185). Thus, investigation into the role of senescence in glucocorticoid resistance may provide novel approaches for the treatment of asthma. In addition, although current studies suggest a role of senescence in asthma, little is known about the pathway regarding the environmental pollutants/allergens, ROS, mitochondrial dysfunction, and senescence. Furthermore, it is poorly understood about the mechanistic links between cell senescence and asthma pathophysiology. Additionally, asthma is typically associated with an imbalance between Th1 and Th2 pathways, and over-driven Th2-mediated inflammation can result in airway inflammation and asthma (186). On the other hand, immune-senescence has been associated with lung aging, and that altered Th1/Th2 imbalance may contribute to the process of accelerated lung aging and immune-senescence (187). Indeed, with aging, mouse lungs showed typically increased Th1 cells with increased levels of IFN-gamma. However, the link between Th1/Th2 cells and senescence remains largely un-explored. Thus, further research is needed to establish the mechanistic links between increased cytokines with aging and senescent cell induction, and asthma pathophysiology.

## Autophagy/Mitophagy and Cellular Senescence

Autophagy plays a role in homeostatic energy supply and elimination of aggregate-prone proteins, damaged organelles, and intracellular microbes. Autophagy also plays a critical role in the regulation of innate and adaptive immune responses in response to environmental stresses. In contrast, cellular senescence is caused by insufficient regulatory mechanisms of homeostasis. One of the most common causes for cellular senescence is that mitochondrial dysfunction results in cellular senescence due to excessive ROS production (153). Although autophagy and senescence are known to share similar properties, recent studies suggest a “double-edged” sword that autophagy can either accelerate the development of senescence or prevent senescence (188, 189). Especially, autophagy can produce large amounts of recycled amino acids, which trigger the production of SASP (e.g., IL-6, IL-8) through the activation of mTOR, thereby leading to senescence (190). By contrast, inhibition of autophagy or insufficient autophagy may promote cell senescence (190). Recent studies suggest that autophagy could be either pro-senescent or anti-senescent, depending on the type of autophagy (general or selective), stimulatory signals, and can be cell-type specific (189). Although it seems that autophagy and senescence are highly related, a great deal of questions remain unanswered regarding the mutual relationship between autophagy and senescence at both molecular and cellular levels in diseases like asthma.

## Therapeutic Interventions

Autophagy, mitophagy, and cellular senescence are potential targets that can be manipulated at various levels, and inhibition of these processes have been considered as potential therapeutic strategies. For example, Liu et al. found that 3-methyladenine (3-MA), an inhibitor of autophagy, suppresses the formation of autophagosomes through the inhibition of PI3K (36). Chloroquine (CQ), another inhibitor of autophagy, also has the ability to inhibit HDM-induced airway remodeling through modulating autophagy pathways (191). In addition, bafilomycine (Baf-A), a macrolide antibiotic derived from *Streptomyces griseus*, can block late-phase autophagy through significant cytosolic acidification (192). In addition, administration of drugs currently in use for asthma (e.g., dexamethasone, montelukast, anti-IL-5, and anti-IgE antibody) can also inhibit autophagy (36). However, these current drugs for autophagy are far from specific, and may play a dual role in modulation of autophagy. Thus, addressing the real impact of autophagy in activation or inhibition of inflammation in disease models is challenging.

It has also been suggested that targeting mitophagy may possess therapeutic potential. Rapamycin and metformin as general autophagy-inducing drugs have been shown to attenuate AMPK and mTOR activity, and preserve energy metabolism through regulating mitophagy and mitochondrial biogenesis stimulation (193, 194). Of note, administration of metformin can induce mitophagy by promoting Parkin activity through p53 downregulation. In addition, several naturally occurring compounds, such as spermidine, resveratrol, urolithin A and antibiotics, have been demonstrated to maintain mitochondrial integrity by the induction of mitophagy and promotion of mitochondrial biogenesis (137). However, the therapeutic potential in human diseases still remains to be determined. Thus, identification of mitophagy modulators may result in therapeutic intervention strategies by targeting mitochondrial-associated pathologenesis of diseases.

Lastly, there is ongoing research to target senescence in cases of pulmonary fibrosis and asthma (195). Specifically, patients with age-related lung diseases (such as COPD and asthma) showed high levels of oxidative stress in the lung tissues, Thus, patients with COPD or asthma could benefit from the use of antioxidants (e.g., NAC, Nrf2 activators, NOX-4 inhibitors, MitoQ), which suppress inflammation and reduce the progression of senescence-associated pathways (196). Moreover, orchestrating SASP modulation could be a better strategy. Indeed, rapamycin, metformin, sirtuin activators, or PAI-1 inhibitor have been suggested to have beneficial effects due to their ability to act as a SASP suppressor (160, 181). For example, mTOR activation has been shown to be essential for asthma onset (197), and inhibition of mTOR with rapamycin can suppress IL-1 translation and reduce mRNA stability of SASP factors (198). Similarly, metformin can also inhibit mTOR and could have the potential to inhibit SASP in asthma. Furthermore, specific induction of apoptosis in senescent cells using senolytics could also lead to beneficial effects (175). Additionally, inducing senescence cell clearance [e.g., ABT-263, also known as navitoclax (199)] by manipulating the immune system to recognize and clear these cells is also an important therapeutic approach. However, these current drugs are far from specific, and some of them may have off-target effects. For example, metformin and sirt1 are also complex I inhibitor, which can promote mitochondrial fission and ROS production and subsequently cell senescence. Furthermore, although SASP is critical for the immune-mediated clearance of senescent cells, it also contributes to tissue dysfunction. Similarly, accumulation of senescent cells with time may lead to age-related loss of structure and tissue function (160, 200). By contrast, senescence can be beneficial in inhibiting the proliferation of transformed cells, and in some of key biological processes such as tissue repairing and wound healing (164). Because of these opposing effects, further studies are clearly needed to understand the exact role of senescence in diseases. Particularly, understanding of the molecular mechanisms regarding the processes involved in senescence will be helpful for the identification of modulators of cellular senescence, which could serve as therapeutic targets for senescence-associated diseases in the future.

## Conclusions

**Figure 4:**
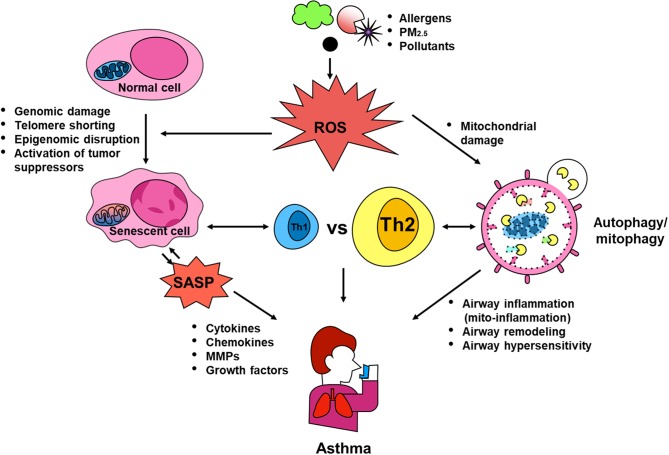
Contribution of autophagy, mitophagy, and senescence to asthma. Environmental triggers, such as environmental pollutants and allergens, can induce excessive ROS generation, which serve as “signaling molecules” modulating the process of autophagic cycle through activating signaling molecules and autophagy, thereby leading to the major phenotypic changes of asthma, including airway inflammation, airway remodeling, and airway hyper-responsiveness. These elevated ROS levels can also induce mitochondrial damage and mitophagy, thereby leading to the mitochondria-induced inflammation (mito-inflammation). Oxidative stress can cause DNA damage, telomere shortening, and epigenomic disruption, which convert normal cells into senescent cells, leading to secretion of SASP. SASP can regulate sensecent cells and induce airway inflammation and remodeling thought the secretion of cytokines, chemokines, MMPs, and growth factors. Additionally, Th1 or Th2 cytokines may induce autophagy/mitophagy/senescence, and in turn, these may also regulate the balance of Th1 and Th2 responses in asthma.

Dysregulations in autophagy, mitophagy, and cellular senescence have been associated with environmental pollutant/allergen-induced oxidative stress, mitochondrial dysfunction, secretion of multiple inflammatory proteins known as SASP, and development of asthma. PM_2.5_ was reported to induce autophagy through activating AMPK (107), and to drive the mitophagy through activating PINK/Parkin pathway (132). PM_2.5_ was also shown to induce senescence of human dermal fibroblasts (177), while increased PM_2.5_ exposure was correlated with shortened telomeres in placental tissue and umbilical blood (178). Intriguingly, allergens, including OVA (36), *A. alternata* (48), and cockroach allergen (47, 111), have also been shown to induce autophagy through activating different signaling pathways. Thus, as hypothesized in Figure 4, environmental triggers, e.g., environmental pollutants or allergens, can induce ROS generation, which serve as “signaling molecules” modulating the process of autophagy through activating downstream signaling molecules and autophagy, thereby leading to the major phenotypic changes of asthma, including airway inflammation, airway remodeling, and airway hyper-responsiveness. These elevated ROS levels can also induce mitochondrial damage, thereby leading to the mitochondria-induced inflammation (mito-inflammation) and major features of asthma. Furthermore, oxidative stress can also cause DNA damage, telomere shortening, and epigenomic disruption, all of which induce cell cycle arrest and cellular senescence. Senescent cells can secrete SASP, which contains multiple inflammatory cytokines, chemokines, mtrix metalloproteinases (MMPs), and growth factors. The SASP leads to airway inflammation and remodeling. In turn, the SASP can also induce senescence and senescent cells can secrete ROS, which further promote the process of senescence. In addition, Th1 or Th2 cytokines may induce autophagy/mitophagy/senescence, and in turn, this autophagy/mitophagy/ senescence may also regulate the balance of Th1 and Th2 responses in asthma. Although comprehensive studies have been focused on investigating the role of autophagy, mitophagy and cellular senescence in the pathogenesis of diseases (e.g., beneficial or detrimental), many questions still remain untouched and unanswered. Thus, future studies are clearly needed to better understand these cellular processes, particularly after exposure to environmental pollutants and allergens, and to identify the therapeutic targets to regulate the autophagy/mitophagy/senescence-associated asthma.

## Author Contributions

KS, YZ, XH, and JC wrote the manuscript. DD and PG reviewed the manuscript.

### Conflict of Interest

The authors declare that the research was conducted in the absence of any commercial or financial relationships that could be construed as a potential conflict of interest.

## Abbreviations

CRE: Cockroach extract
HDM: House dust mite
PM_2._5: Particulate matter 2.5
SASP: Senescence-associated secretory phenotype
ROS: Reactive oxygen species
NAC: N-Acetyl Cysteine
AMPK: AMP-activated protein kinase
PINK1: PTEN-induced kinase
PI3K: Phosphatidylinositol-4,5-bisphosphate 3-kinases
mTOR: Mechanistic target of rapamycin
ATG: Autophagy-related gene
CaMKII: Calmodulin-dependent protein kinase II
CQ: Chloroquine
BAL: Bronchoalveolar lavage
AHR: Airway hyperresponsiveness
COPD: Chronic obstructive pulmonary disease
IPF: Idiopathic pulmonary fibrosis
3-MA: 3-Methyladenine.
